# BRADS and BRWDS: Multipurpose audio and text datasets for automatic Bangla regional speech recognition

**DOI:** 10.1016/j.dib.2025.112177

**Published:** 2025-10-15

**Authors:** Umme Aiman, Md Nakibul Islam, MD Hana Sultan Chowdhury, Md. Sadekur Rahman, Md. Tarek Habib, Mahady Hasan

**Affiliations:** a Department of Computer Science and Engineering, Independent University, Bangladesh; b FabLab IUB, Independent University, Bangladesh; c Daffodil International University, Bangladesh

**Keywords:** Natural language processing (NLP), Bengali language processing (BLP), Automatic speech recognition (ASR), Bangla regional speech recognition, Linguistic diversity, Regional pronunciations, Real-world speech recognition, Bangladeshi dialects

## Abstract

This paper presents an innovative approach to Bangla voice recognition. Although Bangla is the seventh most spoken native language globally, it remains underrepresented in voice recognition research. The dataset contains 298 frequently used Bangla words, including 233 regional words and 65 standard Bangla words. These terms, encompassing various regional pronunciations and meanings, were collected from native speakers in Dhaka, Chattogram, Barisal, Mymensingh, Rajshahi, Sylhet, Rangpur, and Khulna. The 2439 audio segments in the dataset were contributed voluntarily by 85 native speakers and assessed by ten university students. This resource is intended for researchers working on automatic Bangla regional speech recognition systems, with an emphasis on capturing regional pronunciation and linguistic differences. The dataset allows researchers to recreate real-world scenarios during model training by incorporating background noise. Additionally, its modular construction enables further expansion to include new regional words. This multipurpose dataset addresses a critical gap in Bangla speech recognition research and has the potential to drive significant advancements in natural language processing (NLP), particularly with regard to linguistic diversity in Bangladesh.

Specifications Table

**Table:** 

Subject	Artificial Intelligence
Specific subject area	NLP, Signal Processing, Data related to nature, Human Voice, Bengali Regional language and Linguistics
Data format	.wav and .xlsv
Type of data	Audio and Text
Data collection	An effective data-collection technique was developed to gather data systematically and uniformly. Participants were required to record in a .wav file and pause for approximately one second between words during the audio recording process. Damaged or unusable recordings that could not be processed by the splitting technique were removed to maintain the dataset’s integrity. Good data were distinguished from insufficient data by two criteria: incorrectly segmented words (e.g., two or three words that were grouped together) were discarded, and mispronounced words were excluded. Only words that were correctly separated by the splitting algorithm and correctly pronounced by participants were retained for inclusion. A script containing 298 essential Bengali words was created, and the terms were collected from locals in specific locations. Eight local volunteers gathered the vocabulary in each area, and the general public was surveyed using a Google Form and email. In both cases, participants supplied their information and audio files. Participants were instructed to use “Easy Recorder” for Android, “Hokusai 2″ for iOS, and “Raw Recorder” for web applications to record their audio.
Data source location	Institution: Independent University, Bangladesh (IUB), City/Town/Region: Dhaka, Country: Bangladesh, GPS Coordinates: 23.8155° N, 90.4275° E
Data accessibility	The dataset is available on Mendeley. Licensing: Creative Commons Attribution 4.0 International (CC BY 4.0) Text Data: Mendeley Data (DOI: 10.17632/6pd2c48m66.4) Voice Data: Mendeley Data (DOI: 10.17632/33khhwbhwn.2).

## 1 Value of the Data

• This dataset is the first and only open-source Bangla speech corpus explicitly designed to capture regional pronunciation across all eight divisions of Bangladesh. By providing 2439 high-quality WAV recordings of 298 words from Dhaka, Chattogram, Barisal, Mymensingh, Rajshahi, Sylhet, Rangpur, and Khulna, it fills a critical void in dialect-sensitive language resources. Its value lies in its ability to support the development and evaluation of dialect-aware ASR and NLP models.
• Researchers can leverage these recordings to develop and evaluate dialect-aware ASR and NLP models. The division- and word-level annotations support training methods that accurately identify regional variants, which enhances performance compared to using standard corpora alone.
• This dataset also supports the development of machine translation engines, text-to-speech systems, and more inclusive conversational bots. By incorporating authentic regional accents and real-world background acoustics, downstream applications can deliver more natural and engaging interactions in colloquial Bangla contexts. This real-world relevance could significantly improve user experiences across various language technologies.
• The corpus preserves Bangladesh’s linguistic diversity. Featuring 233 region-specific variants alongside 65 standard terms, it serves as both a computational dataset and a sociolinguistic archive, ensuring that under-documented pronunciations remain available for phonetic and cultural studies.

## 2 Background

Research on word detection in low-resource languages such as Bengali remains limited. However, several initiatives have emerged, primarily focusing on recognizing dialectal variations and local lexicons in both spoken and written Bangla. One notable contribution is SUBESCO [1], an audio-only emotional speech corpus that supports cognitive psychology research on emotional expression and prosodic speech analysis. Additional benchmark datasets have been developed for tasks such as news audio classification. The BAAD dataset [2], for example, contains audio recordings of Bangla slang, aimed at minimizing exposure to offensive content in media and safeguarding vulnerable audiences. Another study introduced an autonomous Bangla real-number recognizer using CMU Sphinx 4 and Avro, a widely used Bangla Unicode writing tool [3]. To address challenges with noisy data, researchers proposed continuous word segmentation methods tailored for Bengali noisy speech [4]. Recent additions include KBES, an emotional speech dataset comprising 900 recordings from 35 actors [5], and BanglaDialecto, developed for dialect-aware ASR and translation between the Noakhali dialect and Standard Bangla [6]. Despite these advances, there remains a lack of datasets that comprehensively capture regional Bangla dialects for voice recognition. The BRADS and BRWDS datasets directly address this gap by systematically collecting dialect-rich speech samples from all eight administrative divisions of Bangladesh. Table 1 provides a comparative overview clearly highlighting the key differences between the BRADS & BRWDS dataset and other relevant Bangla speech resources.

**Table 1:** Comparative analysis of Bangla speech datasets.

Dataset name	Speakers	Regional coverage	Dialect diversity	Data type	Application domain
BRADS & BRWDS (this dataset)	85	All 8 divisions	Yes	Audio and text	General ASR & NLP
SUBESCO [1]	50	Limited (Dhaka)	No	Audio (emotional)	Emotional analysis
BAAD [2]	30	Limited (Dhaka)	No	Audio (offensive)	Offensive detection
Continuous Word Segmentation [4]	N/A	Limited	No	Audio (noisy speech)	Speech segmentation methods
KBES [5]	35	Not specified	No	Audio (Emotion)	Speech recognition
BanglaDialecto [6]	N/A	Single dialect	Yes (Noakhali)	Audio and text	Dialect-aware ASR & translation

## 3 Data Description

The dataset created for automatic Bangla Regional Speech Recognition consists of 298 commonly used Bangla words, divided into 65 standard Bangla words and 233 regional variants. The term “Standard Bangla” primarily reflects the usage prevalent in Dhaka, making it the chosen representative for this category. Words were collected from 8 distinct areas in Bangladesh: Dhaka, Chattogram, Barishal, Mymensingh, Rajshahi, Sylhet, Rangpur, and Khulna. Due to the substantial lexical diversity in Chattogram, 80 separate voice folders were maintained to account for multiple word variants. The dataset captures the nuanced pronunciations and meanings of familiar words spoken by rural communities across diverse regions of Bangladesh. Comprising 2439 audio clips, the dataset reflects the collaborative efforts of 85 native speakers, as well as input from 10 different university students for evaluation. It is valuable for training machine learning and AI models to recognize spoken Bangla accurately, including local dialects and colloquial terms. The dataset aims to capture the subtle pronunciations and meanings of these words spoken by individuals from rural communities across diverse regions of Bangladesh. This can lead to the development of more robust voice recognition applications and machines effectively interacting with Bangla speakers.

**Fig. 1:**
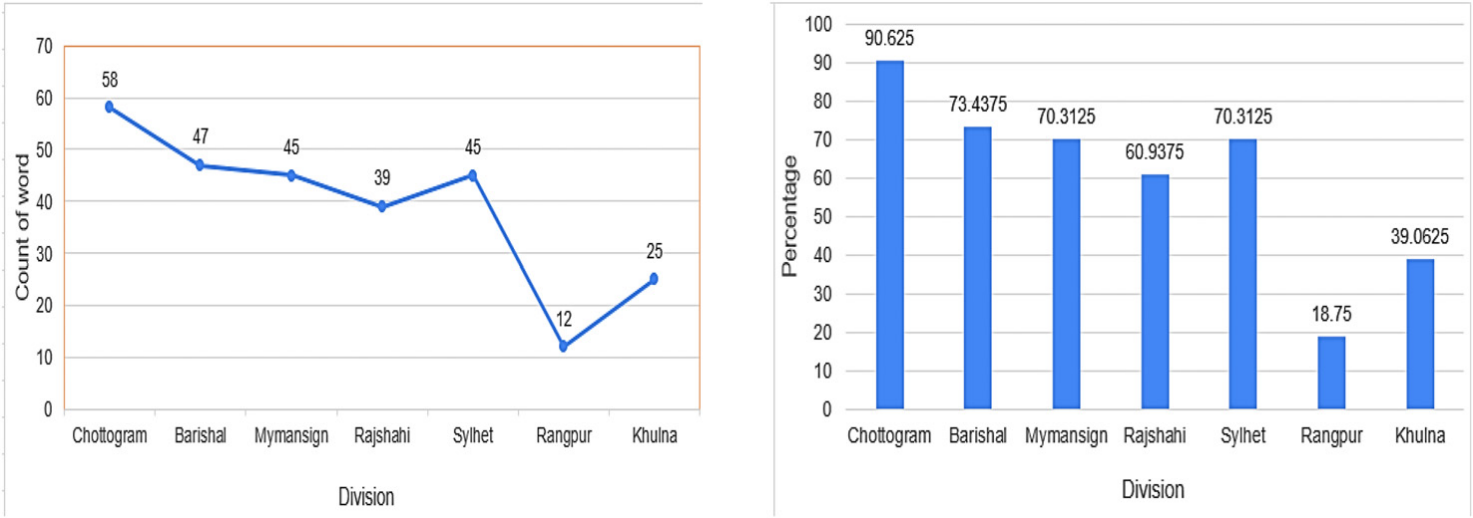
Diversion rate of linguistic diversity across divisions compared to Dhaka.

As illustrated in Fig. 1, the analysis highlights significant lexical variation across divisions. It compares 64 commonly used words from Dhaka with corresponding vocabulary in the seven other divisions. The first graph shows the number of words retained in each region, while the second graph presents the percentage of lexical diversity. Chattogram demonstrates the highest diversity rate, while Rangpur shows the lowest, indicating stronger similarity to Dhaka’s vocabulary.

The variations in lexical choices for a standard term across divisions is demonstrated in Table 2. For example, in Bangladesh, the first-person singular pronoun “” (Ami), meaning “me”, is predominantly used in the Dhaka division, as well as in Sylhet, Khulna, and Mymensingh, whereas “” (Mui) is frequently used in the Barisal division. Similarly, “” (Ayi) is commonly observed in Chattogram, “” (Amak) in Rajshahi, and “” (Hami) in Rangpur. Although they share the same meaning, these variants create distinct regional identities by reflecting historical and cultural experiences. These observations underscore that Bangladesh’s linguistic landscape is dynamic, with dialectal variations changing over short geographic distances.

**Table 2:** Regional variations of pronunciation across divisions.

Including local language words significantly enhances the natural language processing capabilities of various applications. Chatbots can engage more naturally with Bangla speakers, text-to-speech systems produce authentic Bangla speech, and machine translation systems translate accurately between Bangla and other languages.

This dataset helps preserve Bangla's rich linguistic diversity, capturing local variations and nuances otherwise at risk of being lost. It supports research and education, promoting a deeper understanding of Bangla language and culture. Additionally, it can underpin innovative language-learning tools, assisting learners at all levels to better understand and use local Bangla language.

Despite these advancements, a significant research gap remains concerning voice recognition in conjunction with identifying regional vocabulary and Bengali dialects. Researchers examining regional variations in Bangla speech or speech patterns will find this dataset helpful, particularly in training Bangla voice recognition models using machine or deep learning approaches.

The 298 Bangla words in the 'Bangla RDS.xlsv' file were provided to participants during voice recording. This files includes two worksheets. The first sheet, “region wise data”, arranges 298 words across 65 rows and eight columns, with each column representing one of Bangladesh’s eight divisions (Dhaka, Chattogram, Barishal, Mymensingh, Rajshahi, Sylhet, Rangpur, and Khulna). Each row corresponds to a common concept, and the cell entries contain the local variants spoken in the respective division. Participants were instructed to read only the column for their own division, ensuring that each regional variant was captured. The second sheet, “categorize data”, reorganizes the same words by grouping synonyms and standard forms to facilitate cross‑dialect comparison and translation. All words are written in Bangla script (UTF‑8). This workbook allows researchers to map regional vocabulary to Standard Bangla and generate word lists for ASR or translation models.

**Fig. 2:**
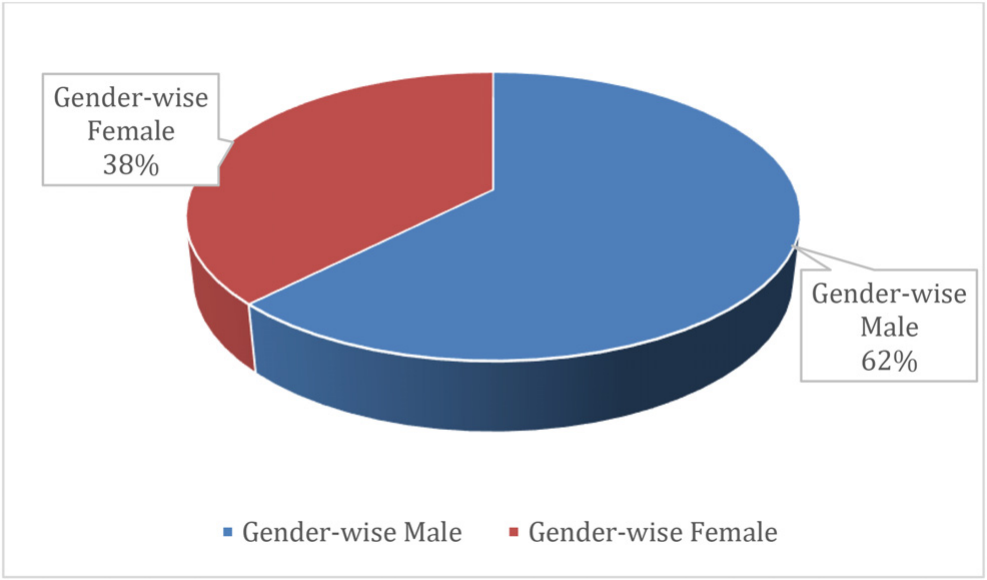
Male-to-female ratio of the audio dataset.

All 298 regional words were manually gathered from local people in their respective regions. Fig. 2 shows that 62 % of participants are male and 38 % are female. Female recordings were retained because higher‑pitched voices enrich the acoustic diversity of the corpus and are important for training models that generalize across speakers. We acknowledge the current gender imbalance and will prioritize recruiting more female participants in future data collection.

To provide a clear overview of speaker demographics and recording conditions, Table 3 summarizes the age groups, gender distribution, divisional coverage, devices and recording environments for each cohort. This structured metadata table follows best practices recommended in dialectal speech datasets [7].

**Table 3:** Speaker metadata for the BRADS and BRWDS datasets.

Gender	Age group	Division	Recording tool	Participant	Environment
Male / Female	18–57	Dhaka	Easy Recorder (Android) / Hokusai 2 (iOS) / Raw Recorder (Web)	10	Quiet indoor
		Chattogram	Easy Recorder / Hokusai 2 / Raw Recorder	13	
		Barisal	Easy Recorder / Hokusai 2 / Raw Recorder	11	
		Rangpur	Easy Recorder / Hokusai 2 / Raw Recorder	11	
		Sylhet	Easy Recorder / Hokusai 2 / Raw Recorder	10	
		Khulna	Easy Recorder / Hokusai 2 / Raw Recorder	10	
		Rajshahi	Easy Recorder / Hokusai 2 / Raw Recorder	10	
		Mymensingh	Easy Recorder / Hokusai 2 / Raw Recorder	10	

**Fig. 3:**
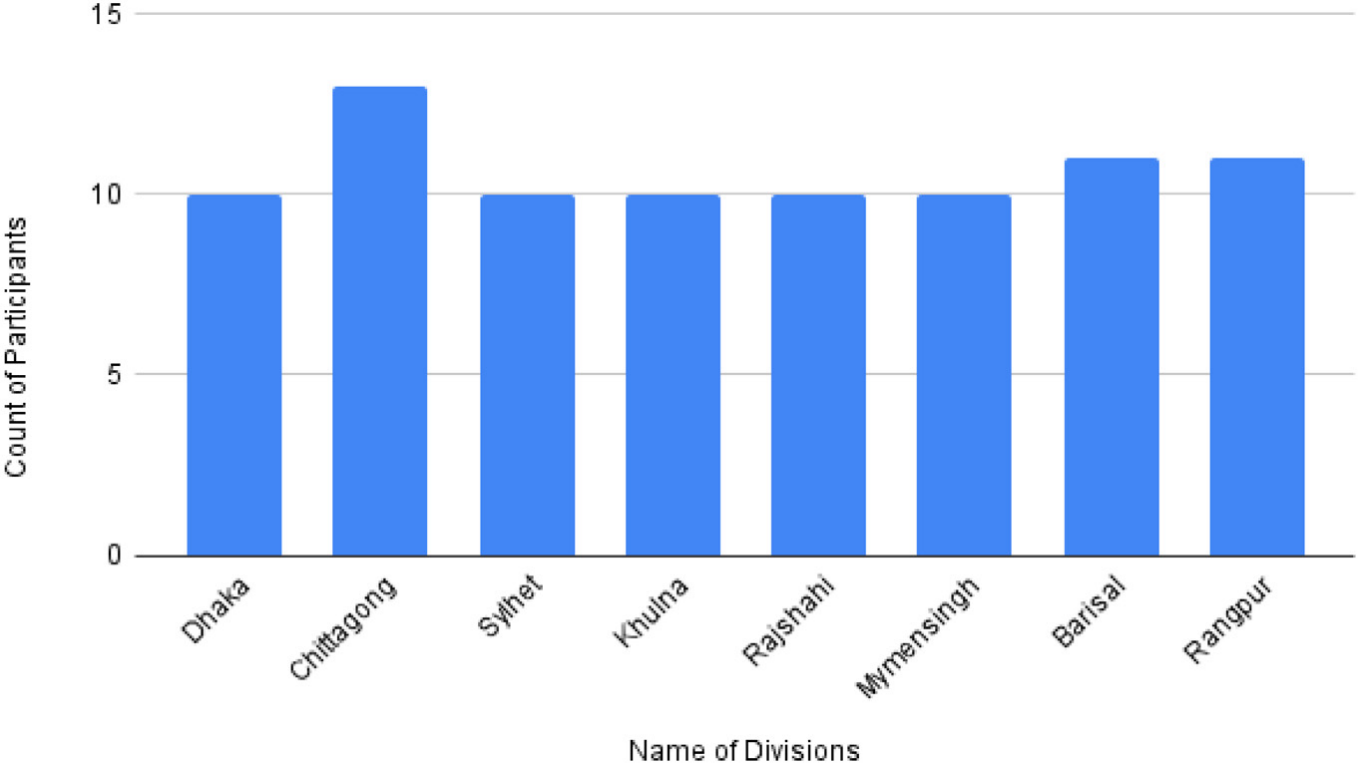
Division-wise regional count of data collection*.*

Fig. 3 displays division-wise regional data collection based on the native dialect. Audio data were gathered from eight districts representing diverse dialects. At least ten participants provided voice data from Dhaka, Sylhet, Khulna, Rajshahi, and Mymensingh divisions; Chattogram had 13 participants, while Barishal and Rangpur divisions had 11 participants each. Multiple participants contributed to other divisions on numerous occasions.

**Fig. 4:**
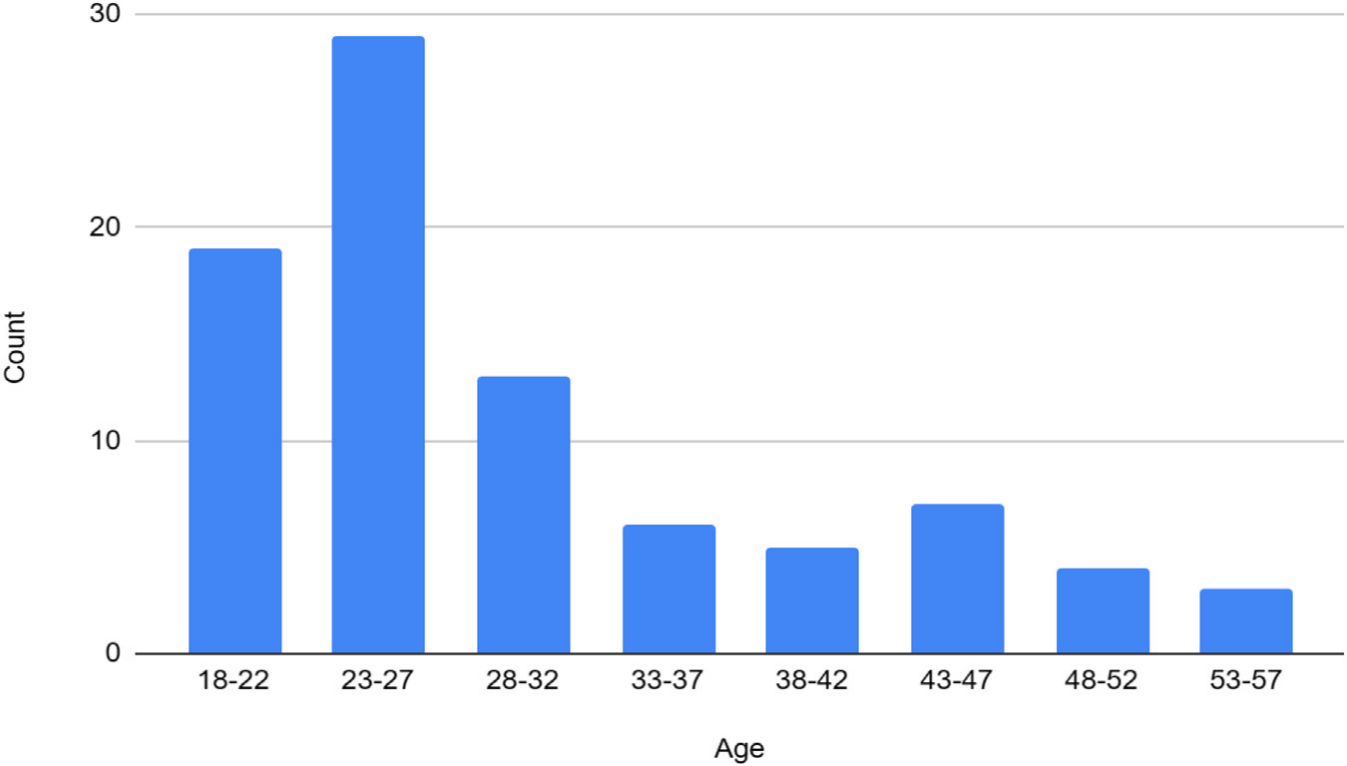
Frequency of age-wise data collected.

The bar chart in Fig. 4 shows the distribution of voice data by age groups. Ages 23–27 provided the most recordings, followed by 18–22 and 28–32. Contributions decrease in older groups, with the fewest from ages 53–57. This highlights younger individuals’ higher engagement, particularly those in their twenties.

To provide a concise overview of participant demographics and recording conditions, Table 3 summarises the age groups, gender distribution, divisions represented, recording devices or apps, environments, and the number of speakers in each group. By consolidating details that are otherwise dispersed across the text, this table makes the dataset composition transparent and replicable.

## 4 Experimental Design, Materials and Methods

### 4.1 Participants and sampling

**Fig. 5:**
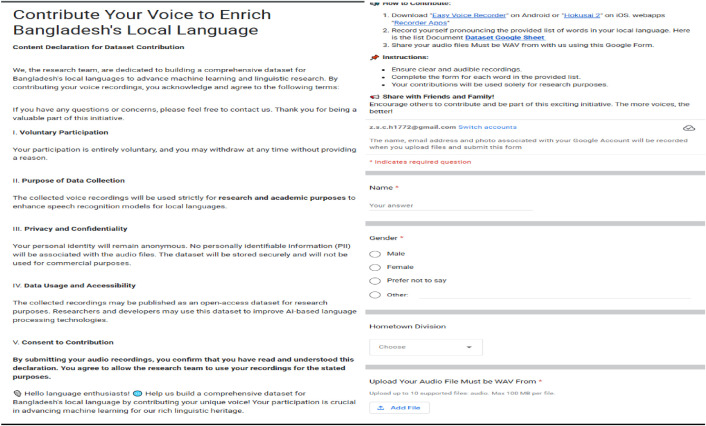
Google form for participant recruitment and data submission; *(Left) consent and information declaration section; (Right) demographic details and WAV audio upload interface.*

Data were collected using a hybrid convenience-and-purposive sampling strategy, in which participation requests were disseminated via social media, university mailing lists, and a publicly accessible Google Form Fig. 5. The form comprised two sequential sections:

• Consent and Information Declaration: Participants first reviewed and acknowledged a consent declaration outlining the voluntary nature of the study, data usage, confidentiality safeguards, and their right to withdraw at any time. This consent process was aligned with ethical protocols reviewed and approved by the university ethics committees.
• Demographic & Audio Submission: In the second section, respondents provided their name, age, gender, email address, and home division via dropdown menus and then uploaded their WAV-format voice recording (maximum 100 MB). Demographic metadata was captured for transparency and future analysis.

We recognize that this self-selected sample**,** driven by voluntary participation, may not yield a fully representative cross-section of all Bangla speakers across the eight divisions. As a result, the sample is skewed toward individuals aged 23–27 years (the majority) and comprises 62 % male and 38 % female respondents. Although these demographic imbalances limit the external validity of the dataset, it nevertheless represents a pioneering resource for regional Bangla speech research.

### 4.2 Data collection protocol

**Fig. 6:**
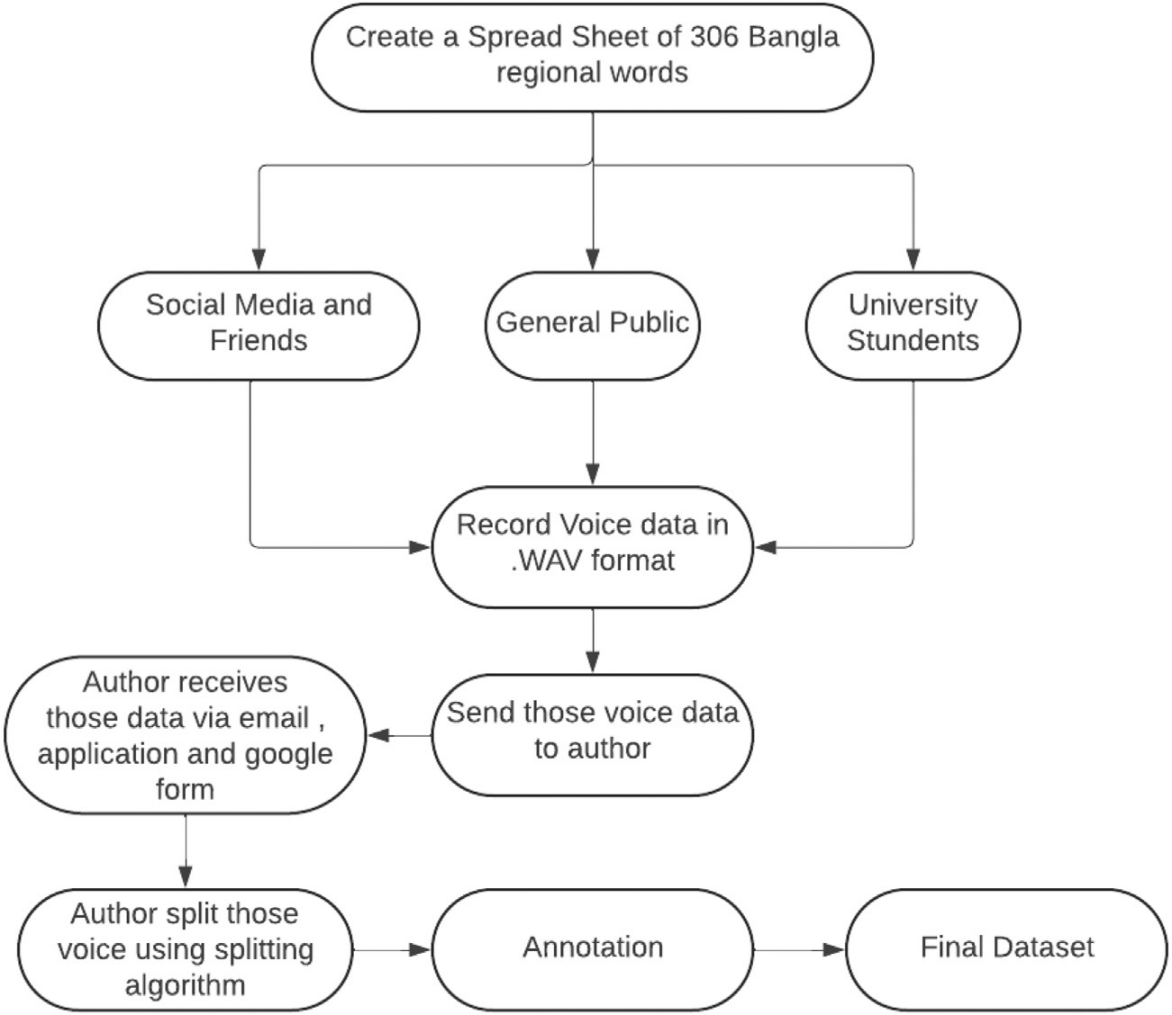
Workflow for the entire voice dataset preparation.

Our voice dataset preparation workflow Fig. 6 begins with a dynamic, web-based data-gathering interface. A custom-designed Google Form was used to collect participant metadata (name, gender, age, and hometown) along with raw audio recordings. The hometown field features a dropdown of Bangladesh’s eight administrative divisions (Dhaka, Rajshahi, Rangpur, Sylhet, Chattogram, Barisal, Khulna, Mymensingh), and participants were instructed to download the attached Bangla RDS.xlsv containing 298 target words (65 words per division).

Participants were prompted to read each word at a rate of approximately three seconds per item, record in a quiet environment (ambient noise ≤ 35 dBFS), and save the audio as 16-bit PCM WAV at 44.1 kHz. Recording tools included: Easy Recorder (v2.1.3) for Android, Hokusai 2 for iOS, and a custom-built web tool called Raw Recorder, optimized for high-quality WAV output.

**Fig. 7:**
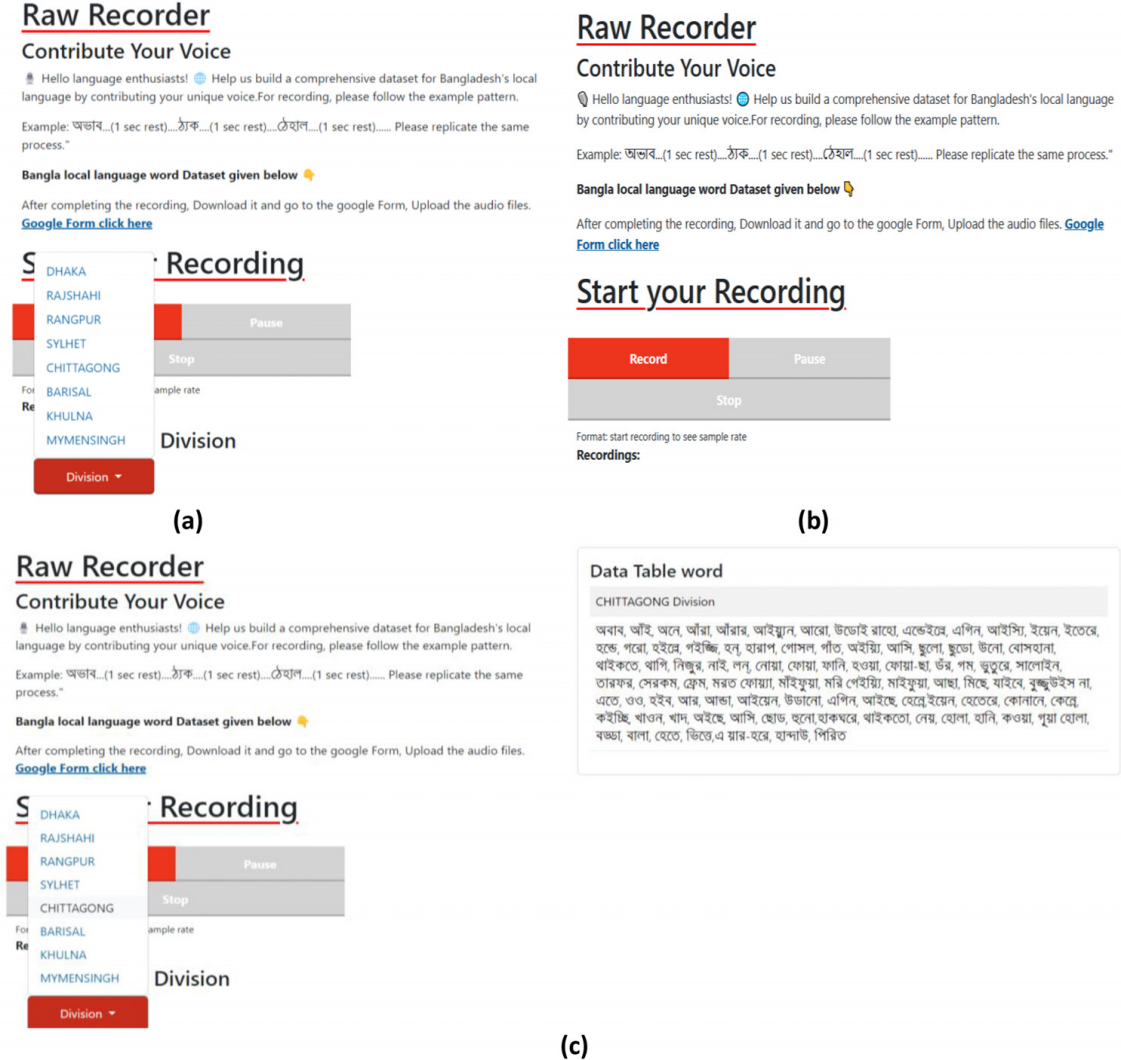
User interface of the raw recorder website; (a) The division can be selected, and the user can record audio; (b) The division can be chosen by clicking on the division dropdown button; (c) Once the designated division is selected from the dropdown, a data table will be displayed on the right side of the web page.

On the Raw Recorder site Fig. 7, users selected their division, viewed a dynamically updated word list based on local dialect, recorded each clip, and submitted their files via the Google Form. Over two weeks, 120 participants across all divisions contributed 3280 raw recordings.

### 4.3 Data segmentation

Each submitted words.wav file is automatically processed using a recursive, silence-based splitting algorithm adapted from Md. et al. [1]. The goal was to generate exactly 298 individual word segments per user.

**Fig. 8:**
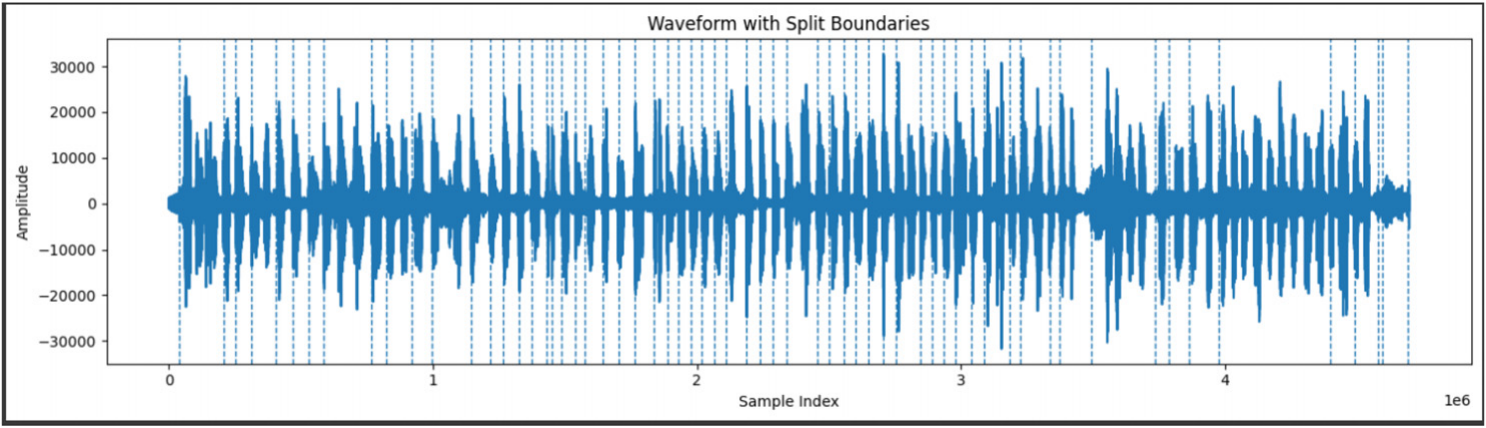
Waveform with segmentation boundaries.

The segmentation process was implemented using the PyDub Python library (v0.25.1), with initial parameters set to a minimum silence length of 300 ms, a silence threshold of –32 dBFS, and 100 ms of silence padding at each boundary shown in Fig. 8. If the output contained more or fewer than 298 segments, the algorithm dynamically adjusted the thresholds by incrementing the silence length by 10 ms and lowering the threshold by 1 dB in each iteration. This adjustment was repeated up to ten times until the segmentation matched the target number of word clips. This adaptive method ensured consistent and language‑agnostic tokenization across diverse audio recording conditions. Beyond silence‑based segmentation, we relied on open‑source tools to inspect and refine the audio. Audacity was used to edit and visualize waveforms and spectral characteristics, while Matplotlib was used in Python to plot segmentation boundaries and quality‑control charts. Algorithm 1 below outlines the logic used for this segmentation approach.

**Algorithm 1:** Recursive audio splitting for tokenizing.

Input: A raw audio file as input, A
Output: Segmented audio clips, B
Step 1: **FOR**
Step 2: Set minimum silence length, *n*
Step 3: Set silence threshold volume, *m*
Step 4: Duplicate the original audio to file *c*
Step 5: Split c using silence length
Step 6–10: For each segment, detect silence, and save start times
Step 11: Calculate non-silent ranges from saved indices
Step 12: If non-silent range count = 298 → accept segmentation
Step 13: Else, adjust n and m, repeat
Step 14: BREAK once count matches

### 4.4 Data annotation

After segmentation, clips were organized into a two‑level folder structure, one folder per division, each containing 298 subfolders by word ID. Three trained annotators (Umme A., Md. N. I., and Md. H. C.) independently reviewed all clips. Correctly pronounced samples were moved to their respective word folders, while mispronounced or noisy clips were transferred to a “Waste Data” folder. To ensure annotation consistency, we calculated inter‑annotator agreement on a 10 % random sample, yielding Cohen’s κ = 0.88. Of the initial 3280 segments, 841 (25.6 %) were rejected due to pronunciation issues or corruption, resulting in 2439 validated, high‑quality recordings.

## Limitations

While the words were collected, significant challenges were encountered in identifying and verifying that the correct native speakers were selected for each division. Eight individuals were required for data input to ensure the dataset remained authentic and accurate. Another major limitation was faced during data annotation; after collecting 3280 voice recordings, each piece of data had to be individually reviewed to filter out poor-quality recordings. Additionally, finding participants for each division posed another challenge. To maintain authenticity, the pronunciation styles of each contributor had to be thoroughly verified.

## Ethics Statement

Ethical approval for this study was granted by the Research Ethics Committees of Independent University, Bangladesh – Faculty of Science and Information Technology (Approval No 2025‑NON‑SR‑SETS‑01) and Daffodil International University (Ref: REC‑FSIT/DIU/2024/1023).

Participant recruitment and consent were managed through a structured process using a digital Google Form. Each participant reviewed a consent statement that outlined the purpose of the study—the collection of anonymized Bangla speech samples for dialectal and linguistic research—as well as the voluntary nature of participation, their right to withdraw at any time without consequence, and the ability to request removal of their data after submission. No personal identifiers (such as names or email addresses) were stored alongside the recordings. All demographic metadata (e.g., age, gender, division) was collected with explicit consent and stored in de-identified, aggregated form to ensure participant privacy. The finalized dataset is released under a Creative Commons Attribution 4.0 International (CC BY 4.0) license, allowing open access for academic and research purposes while maintaining transparency and ethical data use. All study procedures, including data collection, segmentation, annotation, and publication, were conducted under the supervision of the Department of Computer Science at Independent University, Bangladesh, with advisory oversight from Daffodil International University’s ethics board. Protocol documentation and anonymized logs have been securely archived for transparency and audit purposes.

## Credit Author Statement

**Umme Aiman:** Writing – Original draft, Data curation, Validation, Investigation; **Nakibul Islam:** Conceptualization, Data curation, Investigation, Software, Resources, Validation; **Md. Hana Sultan Chowdhury:** Conceptualization, Methodology, Software, Resources, Data curation; **Md. Sadekur Rahman:** Visualization, Supervision; **Md. Tarek Habib:** Methodology, Supervision; **Mahady Hasan:** Writing review and editing, Supervision.

## Data Availability Statement

The complete dataset capturing linguistic diversity across Bangladesh’s eight divisions is freely accessible on Mendeley Data, providing researchers and linguists with ready access to both the text data (lexicon) and audio data (corpus).

**Text Data (Lexicon):** Mendeley Data – BRWDS: A Multipurpose Dataset for Bangla Regional Word Detection (DOI: 10.17632/6pd2c48m66.4).

**Voice Data (Audio):** Mendeley Data – BRADS: A Multipurpose Audio Dataset for Bangla Regional Word Detection (DOI: 10.17632/33khhwbhwn.2).

## Data Availability

Mendeley DataBRADS: A Multipurpose Audio Dataset For Bangla Regional Word Detection (Original data)
Mendeley DataBRWDS: A Multipurpose Dataset For Bangla Regional Word Detection (Original data)
